# From gut to lung: the microbiome-directed causal mechanisms and therapeutic horizons in asthma

**DOI:** 10.3389/fimmu.2026.1935791

**Published:** 2026-09-28

**Authors:** Feng-Xian Ni, Chun Feng Pan, Hui-Hui Chen, Dong-Hui Huang, Ze-Bo Jiang

**Affiliations:** 1 Zhuhai Hospital of Integrated Traditional Chinese and Western Medicine, Zhuhai, China; 2 Zhuhai Hospital Affiliated to Faculty of Chinese Medicine, Macau University of Science and Technology, Zhuhai, China

**Keywords:** asthma, causal inference, gut-lung axis, microbiome, multi-omics, precision medicine, regulatory T cells, short-chain fatty acids

## Abstract

The gut–lung axis represents a predominantly gut-to-lung communication network through which gut microbiota and their metabolites influence pulmonary immunity. In asthma, gut dysbiosis—characterized by reduced microbial diversity, depletion of short-chain fatty acid (SCFA)-producing taxa, and expansion of pro-inflammatory pathobionts—has been consistently associated with disease severity and type 2 inflammation. While the axis is conceptually bidirectional, the vast majority of mechanistic evidence to date describes unidirectional gut to lung regulation. This review critically synthesizes evidence on the gut-lung axis in asthma. We evaluate multi-kingdom microbial ecology, evaluating contributions from bacterial, fungal, protozoan, and phage. We examine metabolite-specific immune reprogramming via SCFAs, bile acids, and tryptophan derivatives, explicitly acknowledging unresolved controversies including the SCFA concentration paradox. We integrate causal inference frameworks that combine Mendelian randomization, gnotobiotic models, and experimental triangulation. We also present precision therapeutic pipelines with evidence-based grading of interventions ranging from probiotics to engineered live biotherapeutics. We propose that targeting the gut–lung axis represents a foundational strategy for asthma modification and outline a roadmap from multi-omics discovery to biomarker-guided clinical trials. Distinguishing this review from prior work, we explicitly separate established mechanisms from speculative pathways and provide actionable criteria for clinical translation. We also critically identify the reverse (lung to gut) pathway as a major knowledge gap requiring urgent investigation.

## 1 Introduction

Asthma is a heterogeneous chronic respiratory disease affecting over 300 million people worldwide. Prevalence is rising most rapidly in low- and middle-income countries (GBD 2021) (1). Traditionally viewed as an airway-centric disorder characterized by chronic inflammation, airway hyperresponsiveness, and variable airflow obstruction, asthma is now conceptualized as a systemic condition. This heterogeneity extends to its underlying pathophysiology, encompassing not only the well-characterized type 2 (T2)-high eosinophilic phenotype but also non-T2 endotypes, including neutrophilic, paucigranulocytic, and obesity-associated asthma. These non-T2 phenotypes, which are often more severe and steroid-resistant, are driven by distinct immunological pathways involving Th17 cells, neutrophils, and innate immune activation (2–4). Crosstalk between distant mucosal surfaces—particularly the gut and the lung—plays a decisive role in shaping susceptibility, phenotype, and therapeutic response (5, 6).

The gut microbiota has emerged as a potent, modifiable determinant of asthma pathogenesis. Over the past decade, accumulating evidence has repositioned the gut from a passive antigen reservoir to an active immunological rheostat. This rheostat calibrates immune development and influencing distal organs, including the lung (7, 8). The hygiene hypothesis provided the intellectual foundation for this paradigm shift, linking early-life microbial exposures to reduced allergic disease risk (9). Population-based studies have subsequently associated reduced microbial diversity, urbanization, antibiotic exposure, Caesarean delivery, and diminished environmental contact with increased childhood asthma risk (10–12). High-throughput sequencing has established reproducible associations between specific fecal or airway microbial signatures and asthma phenotypes (13, 14). However, associations do not equate to causation. The field has progressively pivoted from descriptive microbiome cataloguing toward mechanistic interrogation, causal inference, and therapeutic manipulation (15).

The contemporary view of the gut–lung axis is inherently ecological and systems-based. The microbiome constitutes a dynamic, cross-domain ecosystem encompassing bacteria, viruses (including bacteriophages), fungi, archaea, and eukaryotic microbes (16, 17). Critically, the axis operates bidirectionally. In principle, pulmonary inflammation—and the antibiotic treatments used for secondary respiratory infections—can alter gut communities via systemic immune mediators and antibiotic exposure as well as via systemic inflammation-induced changes in gut permeability. Conversely, gut-derived signals—metabolites, microbial components, and immune cells—can remotely modulate pulmonary immunity (5, 18). In practice, however, the literature—and by extension this review—is heavily biased toward the gut–lung axis, with the lung–gut direction being supported largely by indirect evidence, such as systemic inflammatory effects on the intestinal tract, rather than by direct mechanistic validation. Recent breakthrough studies have reshaped our understanding. The demonstration that intestinal fungi can translocate and drive pro-fibrotic pathways in the lung, and that gut-resident protozoa can systemically activate type 2 immune effectors (19), challenges the long-held notion of pulmonary immunological autonomy.

Several comprehensive reviews have described associations between gut dysbiosis and asthma (5, 20, 21). However, the field now requires a framework that moves beyond descriptive cataloging. Four pressing questions remain unanswered. How do multi-kingdom microbes—fungi, protozoa, and phages—collectively and interactively influence asthma pathogenesis beyond bacteria? Through which specific molecular pathways do microbial metabolites remotely program pulmonary immunity, and which mechanisms remain speculative? Which associations represent causal drivers versus epiphenomena, and what experimental triangulation strategies can establish causality in humans? How can mechanistic insights be translated into precision diagnostics and therapeutics with demonstrated clinical utility?

This review directly confronts these questions. We provide a critical synthesis that explicitly distinguishes established mechanisms from emerging hypotheses, evaluates evidence quality using a structured grading system; identifies unresolved controversies including the SCFA concentration paradox; and proposes testable frameworks for causality and translation. By adopting this critical stance, we aim to accelerate the transition from associative microbiome science to mechanism-based precision medicine in asthma.

## 2 The gut microbial ecosystem in asthma: beyond bacteria

### 2.1 Gut dysbiosis in asthma: functional deficits, not taxonomic shifts

Patients with asthma exhibit a distinct and progressive gut dysbiosis that correlates with disease severity. Numerous studies have reported reduced α-diversity and altered taxonomic composition, typically characterized by depletion of beneficial commensals and expansion of pro-inflammatory taxa (22, 23). However, functional metagenomic analyses reveal more consistent deficits than taxonomic shifts alone. Regardless of which specific bacterial species are present or absent, the gut microbiome of asthmatic patients shows a reproducible reduction in the capacity to perform certain metabolic functions. The most robust and reproducible association with asthma severity across independent cohorts is the depletion of SCFA-producing metabolic pathways, rather than the absence of any particular bacterial taxon (20, 24).

This functional perspective offers several important advantages over purely taxonomic approaches. Different bacterial taxa can perform the same function—butyrate production, for instance, is carried out by Faecalibacterium, Roseburia, and Lachnospiraceae. Focusing on functional capacity rather than taxonomic composition therefore reduces inter-study variability and provides clearer mechanistic insights. Moreover, functional deficits represent actionable therapeutic targets: restoring metabolic function may prove more clinically relevant than attempting to reinstate specific bacterial species. Most importantly, functional pathways have defined molecular mechanisms, enabling hypothesis-driven investigation and precise intervention design.

Key functional deficits consistently observed in asthma include reduced butyrate synthesis capacity, evidenced by decreased abundance of butyryl-CoA: acetate CoA-transferase genes—a gene cluster found in butyrate-producing genera including Faecalibacterium, Roseburia, Eubacterium, and Lachnospiraceae (25, 26). Impaired bile acid deconjugation affects secondary bile acid production (27). Reduced tryptophan metabolism leads to lower levels of indole-3-aldehyde and indole-3-propionic acid (28, 29). Concurrently, opportunistic taxa—include Enterobacteriaceae, Candida species, and Haemophilus—may expand and generate pro-inflammatory signals that reach the lung (23, 30). The multi-kingdom contributions to gut-lung axis dysregulation are summarized in **Table 1**.

**Table 1:** Multi-kingdom microbial contributors to gut–lung axis dysregulation in asthma: functional deficits and evidence quality.

Domain	Key functional deficit	Molecular mediators	Immune effects	Evidence quality	References
Bacteria	Reduced butyrate synthesis	↓Butyryl-CoA: acetate CoA-transferase→HDAC activation→reduced Foxp3 acetylation	Impaired Treg differentiation; Th2/Th17 skewing	High (meta-analysis)	(25, 31)
Fungi	Candida overgrowth; translocation	β-glucans, mannans, chitin; Dectin-1/Raf1/ERK signaling	Pro-fibrotic macrophage polarization; Th17/Th2 skewing	Moderate (mouse causal + human associative)	(22, 32)
Protozoa	T. musculis colonization (mouse models)	S1PR1 upregulation on ILC2s	Gut-primed ILC2 migration to lung; amplified type 2 immunity	Moderate (mouse causal; human associative)	(19, 33)
Phages	Caudovirales shifts	Lytic cycles; lysogenic conversion	Modulation of bacterial metabolite production	Emerging (human associative)	(34)
Archaea	Methanobrevibacter abundance changes	Methanogenesis; hydrogen cross-feeding	Altered fermentation efficiency; SCFA modulation	Emerging (human associative)	(35)

*Evidence Quality Definitions: High = meta-analysis or multiple consistent RCTs/cohorts; Moderate = animal models + human associations; Emerging = observational only.

### 2.2 Fungal: translocation and direct pathogenic roles

Although fungi constitute a minor fraction of gut biomass, they carry disproportionate immunomodulatory potential. Their cell walls are rich in β-glucans, mannans and chitin. These components engage dectin-1, dectin-2, Mincle, and Toll-like receptor 2 (TLR2), eliciting potent innate responses (36–39).

Fungi produce unique metabolites that influence epithelial integrity and immune polarization (40). β-glucans signal through Dectin-1 on epithelial and immune cells to modulate barrier function. Mannans can disrupt tight junctions and increase epithelial permeability. Chitin activates inflammatory responses via TLR2 and NOD2. Fungal secondary metabolites such as gliotoxin and fumagillin have immunosuppressive or cytotoxic effects on epithelial cells. These metabolites can act locally in the gut or systemically, contributing to the gut-lung axis (40).

The relationship between gut fungi and epithelial integrity is complex. Chikina et al. reported that in the absence of macrophages protrusions, colonic epithelial cells absorb fluids containing fungal products, leading to barrier loss (41). Conversely, Cao et al. showed that upon *Candida albicans* stimulation, intestinal epithelial cells secrete the histidine methyltransferase METTL9. This enzyme binds the fungal zinc-scavenging protein PRA1 and catalyzes histidine methylation. This disrupts zinc acquisition and restricts fungal colonization—a host defense mechanism that limits Candida overgrowth (42). In the context of asthma, the net effect of this pathway remains unclear. While limiting Candida overgrowth may reduce pro-inflammatory fungal signals, excessive immune activation against fungi could paradoxically exacerbate type 2 inflammation. Further research is needed to determine whether this pathway is protective or harmful in asthma.

Critically, under gut barrier disruption, commensal fungi can translocate to systemic sites including the lung (32). The mechanistic cascade involves translocation of filamentous fungi (e.g., *Engyodontium* spp.) during increased intestinal permeability. These fungi are taken up by alveolar macrophages. This activates the dectin-1–Raf1–MEK–ERK signaling axis, driving a pro-fibrotic macrophage phenotype (32, 43–45). This pathway drives TGF-β1 and collagen gene programs. It promotes subepithelial fibrosis and airway remodeling (46–48).

In human asthma, clinical evidence directly links gut fungal dysbiosis to disease severity. A 2023 study by Kanj and colleagues examined both mice and humans with asthma (22). In the human cohort, increased intestinal *Candida* abundance was independently associated with a higher risk of severe asthma exacerbations in the preceding year. This association remained significant even after adjusting for systemic antibiotic and corticosteroid use. Mechanistically, the mouse arm of the study showed that gut *Candida* dysbiosis increased pulmonary ILC2 abundance. This occurred even in the absence of allergen sensitisation, suggesting that ILC2s may serve as a mechanistic bridge between gut fungi and lung type-2 inflammation.

In patients with severe asthma characterized by prominent airway remodeling, elevated *exogenous* β-glucan exposure and airway fungal colonization with taxa matching gut isolates suggest a shared origin. This points to possible translocation from the gut to the lung (49). However, most translocation data derive from murine models with artificially induced barrier disruption. Whether this occurs in human asthma without overt gut disease remains uncertain. The human association data, while compelling, do not prove causality.

Therapeutic nodes include barrier enhancement with metabolites such as butyrate, which strengthens tight junctions. Antifungal therapy with agents such as fluconazole or echinocandins in cases of confirmed fungal overgrowth or translocation, though routine use in asthma is not recommended without evidence of fungal sensitization or infection. Dectin-1 pathway inhibition using receptor antagonists or downstream signaling inhibitors (e.g., MEK inhibitors), which are being explored preclinically. Restoring bacterial colonization resistance through probiotics (e.g., Lactobacillus, Bifidobacterium strains) or prebiotics that promote growth of commensal bacteria that compete with fungi for nutrients and epithelial adherence sites (47, 50).

### 2.3 Protozoa: gut-primed type 2 immunity

Protozoa and microbial eukaryotes—often overlooked in microbiome surveys—can exert powerful effects on host immunity. These effects transcend local gut niches (51). A pioneering 2025 investigation demonstrated that colonization by the rodent-specific commensal *Tritrichomonas musculis* (*T. mu*) triggers epithelial and myeloid responses. This expands group 2 innate lymphoid cells (ILC2s) in the intestinal lamina propria (22, 52, 53). These ILC2s upregulate sphingosine-1-phosphate receptor 1 (S1PR1), egress via lymphatics and migrate to the lung, where they respond to allergens or epithelial alarmins with exaggerated IL-5 and IL-13 production, amplifying eosinophilic inflammation and airway hyperresponsiveness (54). While T. mu is rodent-specific, this work provides a paradigmatic framework for gut commensals to systemically prime ILC2s (23).

Crucially, T. mu is rodent-specific. Direct translation to human asthma is not established. Nevertheless, human data hint at complex relationships between intestinal protozoa and allergic disease. A study in Egyptian children found that *Blastocystis hominis* was detected in 43.3% of asthmatic children versus 23.3% of non-asthmatic controls. These children had elevated serum complement C3 and IgE levels, suggesting a genotype-dependent pro-allergic potential (33). While direct epidemiological evidence linking overall gut eukaryotic diversity to systemic immune profiles in humans is currently limited, studies on specific protozoa, such as Blastocystis hominis, have hinted at associations with allergic sensitization (33). These findings are observational and cross-sectional, and no study to date has demonstrated that human protozoa can prime ILC2s in the manner of T. mu. However, they underscore the potential for eukaryotic members of the microbiota to influence asthma pathogenesis, and the field urgently needs to move beyond bacterial-centric views to include these organisms in multi-kingdom analyses of human cohorts.

Several key unresolved questions remain. Which eukaryotic taxa in humans possess the capacity to prime ILC2s? What is the longevity of recruited ILC2s in the lung? Are there opposing regulatory eukaryotes that might counterbalance ILC2 priming? Future research should combine longitudinal human cohort studies with deep multi-kingdom sequencing and functional testing in humanised gnotobiotic models (55).

### 2.4 Phages: ecosystem engineering

Bacteriophages dominate the gut virome numerically and exert top-down control over bacterial populations through lytic cycles and lysogenic conversion (56, 57). By shaping bacterial community structure and gene flux, phages alter the prevalence of SCFA producers, bile acid modifiers, and other functionally critical taxa (58). For example, phage-mediated lysis can release bacterial DNA and metabolites into the gut lumen (59), and the resulting phage-bacteria dynamics can drastically alter the metabolic output of the community, including SCFA biosynthesis (60). The lysogenic conversion of SCFA-producing bacteria can also directly alter their fitness and metabolic gene expression, as exemplified by temperate phage-encoded auxiliary metabolic genes involved in pyruvate metabolism that enhance nutrient availability for SCFA production (60). Importantly, the gut virome (phages) can be directly sensed by the host via pathways such as TLR9, independently influencing asthma susceptibility (59).

Longitudinal cohort analyses suggest that shifts in phage community composition can precede detectable changes in bacterial taxa. This implies that virome perturbations may be upstream drivers of microbiome instability (58, 61, 62). For example, Leal Rodríguez et al. demonstrated that the infant gut virome composition—specifically the abundance of Caudovirales phages and their associated bacterial hosts—was associated with preschool asthma risk independently of bacterial composition (34). Similarly, Tisza et al. showed that longitudinal phage-bacteria dynamics in early life could predict later immune development trajectories (63). This suggests that the virome may act as a key regulator of microbiome maturation during critical developmental windows. Furthermore, phages could represent a novel therapeutic tool (phage therapy) to selectively deplete pathogenic bacteria (e.g., Enterobacteriaceae) without causing the broad collateral damage of antibiotics, although this approach remains to be tested in the context of asthma. Early-life phageome trajectories may thus represent biomarkers or mechanistic levers for later asthma risk (64). Technical hurdles remain. These include low viral nucleic acid abundance and vast genomic diversity. These challenges require integrative approaches like viral particle enrichment and long-read sequencing.

### 2.5 Archaeome and other underappreciated domains

Archaea, though less studied, contribute to gut metabolic networks—notably methanogens (e.g., *Methanobrevibacter smithii*). These influence hydrogen transfer and cross-feeding, thereby affecting fermentation pathways and SCFA yields (35, 63). Changes in archaeal abundance—typically increases in Methanobrevibacter smithii—correlate with altered fermentation efficiency. This may contribute to metabolic phenotypes that intersect with obesity-associated asthma. Increased methanogenesis can reduce hydrogen accumulation. This potentially enhances bacterial fermentation efficiency and SCFA yields. However, some studies suggest that excessive methanogen abundance may be associated with increased energy harvest and obesity. This could indirectly exacerbate asthma through metabolic pathways. The net effect on asthma remains unclear and requires further investigation. The functional role of archaea in this context is an emerging area that could reveal new mechanisms for host energy balance and immune modulation. For instance, the cross-feeding interactions between methanogens and bacteria can influence the production of SCFAs, but whether this translates to a clinically meaningful impact on asthma pathogenesis is a key unanswered question that should be addressed in future metabolomic and gnotobiotic studies.

### 2.6 Cross-domain interactomics: principles and analytical frameworks

Cross-domain interactomics explores how taxa across kingdoms interact functionally. Key modalities include metabolic cross-feeding, physical association within multispecies biofilms, horizontal gene exchange, and immune modulation via combined microbial signals. Analytical frameworks for robust cross-domain inference require integrative pipelines involving paired multi-kingdom sequencing, metatranscriptomics, metabolomics, and network analysis combined with causal modeling (65). Methodological advances have been pivotal, including virome enrichment protocols, improved fungal reference databases, culture-based multi-kingdom co-culturing platforms, and single-cell multi-omics coupled with spatial metabolomics.

### 2.7 Translational and clinical evidence

Longitudinal birth and adult cohorts incorporating multi-kingdom profiling have begun to reveal key patterns. Early-life fungal colonization patterns, such as reduced diversity and *Candida* dominance, predict later wheeze and asthma in some cohorts (66, 67). Virome instability in infancy correlates with later allergic sensitization independent of bacterial composition (34, 63). Co-occurrence signatures (e.g., *Candida* with low butyrate producers) are often more predictive of severe asthma phenotypes than any single taxon (68, 69).

Direct manipulation studies in humans, while nascent, are informative. Dietary fermentable fiber interventions can increase SCFA levels and reduce markers of airway inflammation. However, responses vary based on baseline mycobiome and virome states (69, 70). Circulating β-glucan and fungal DNA have been proposed as potential indicators of an invasive mycobiota that correlates with remodeling biomarkers (71).

Given the multi-kingdom complexity, therapeutic strategies should be ecological and precision-targeted. The perinatal and early childhood windows are crucial for intervention. For established disease, combining microbial modulation with biologics may produce synergistic benefits.

## 3 Molecular and cellular pillars of remote communication

Having established the multi-kingdom microbial ecosystem and its alterations in asthma, we now address the fundamental question: how do these microbial communities communicate with the distant lung? This communication occurs through two interconnected channels. Soluble metabolites act as hormone-like signals that travel systemically. Immune cells educated within the gut subsequently traffic to pulmonary tissues, carrying ecological memory from one mucosal surface to another.

### 3.1 Microbial metabolites as systemic immune messengers

Microbial metabolites occupy the interface between ecology and host physiology. These small molecules serve as the primary chemical language through which gut microbes communicate with distal organs. Several metabolite classes have emerged as central mediators of gut–lung communication: short-chain fatty acids (SCFAs), bile acids and their derivatives, tryptophan metabolites that engage aryl hydrocarbon receptor (AhR) signaling, and complex glycoconjugates such as polysaccharide A (PSA) (72, 73). Their activities are interdependent, temporally dynamic, and context-sensitive. Understanding this chemical crosstalk is essential for deciphering how microbial ecology translates into systemic immune modulation. **Table 2** summarizes the key metabolites and their immunomodulatory roles.

**Table 2:** Key microbial metabolites and their immunomodulatory roles in the gut-lung axis.

Metabolite class	Primary microbial source	Receptor/target	Mechanism of action	Immunological effects	Impact on asthma outcomes	References
Short-chain fatty acids (butyrate, propionate, acetate)	**Firmicutes** (*Faecalibacterium, Lachnospiraceae, Roseburia*)	GPR41/43, GPR109a; HDAC (class I/IIa)	HDAC inhibition→ histone hyperacetylation; GPCR-mediated cAMP/PKA signaling	Promotes Treg differentiation (Foxp3 induction); suppresses Th2/Th17 polarization; enhances epithelial barrier (tight junction proteins)	Reduced airway hyperresponsiveness; decreased eosinophilic inflammation; improved asthma control	(31, 74–76)
Secondary bile acids (LCA, DCA, isoalloLCA)	***Clostridium***, ***Eubacterium***, *Bacteroides*	**FXR, TGR5, PXR, AhR**	TGR5→ cAMP/PKA →NF-κB suppression; FXR → SOCS3-mediated STAT inhibition	Induces anti-inflammatory macrophages; modulates Th17/Treg balance	Protective in some contexts; deficiency may impair host defense	(27, 77)
Tryptophan metabolites (indole-3-aldehyde, indole-3-propionic acid)	***Lactobacillus***, ***Bifidobacterium***, *Clostridia*	**Aryl hydrocarbon receptor (AhR)**	**Enhances epithelial** AhR nuclear translocation → CYP1A1 induction; IL-22 transcription	Enhances epithelial barrier integrity (tight junctions, mucin); promotes ILC3/IL-22 response; reduces Th2 cytokine production	Associated with reduced allergic sensitization; lower FeNO in some cohorts	(28, 78)
Polysaccharide A (PSA)	***Bacteroides fragilis***	TLR2 on CD4+ T cells and APCs	TLR2 signaling → NF-κB → IL-10 production; MHC-II-dependent T cell activation	Induces IL-10-producing Foxp3+ Tregs; suppresses Th2/Th17 responses; promotes tolerogenic DCs	Protective in murine asthma models; human studies pending	(79)

Bold values indicate the most robust and reproducible evidence supporting the corresponding metabolite–immune interaction, based on meta-analyses, multiple consistent randomized controlled trials, or convergent human and animal data.

### 3.2 Short-chain fatty acids: epigenetic and metabolic regulators

SCFAs (acetate, propionate, butyrate) are fermentation products of dietary fibers by anaerobic gut bacteria. Notable producers include members of *Clostridia* and *Lachnospiraceae* (80). These metabolites rank among the most extensively studied gut-derived signals in allergic airway disease, and for good reason. Their immunological potency arises from two evolutionarily conserved mechanisms that operate in parallel.

The first mechanism involves ligand-mediated signaling via G-protein coupled receptors. SCFAs bind to GPR41 (also known as FFAR3), GPR43 (FFAR2), and GPR109A on immune cells and epithelial surfaces (81). The second, equally important mechanism involves inhibition of histone deacetylases (HDACs). This leads to chromatin remodeling and altered transcriptional programs across multiple cell types (81). At epithelial interfaces, SCFAs reinforce barrier integrity via increased mucin production and tight junction stabilization, thereby limiting the passage of luminal antigens and microbial products that could trigger pulmonary inflammation (82).

In adaptive immunity, butyrate stands out as a particularly potent inducer of peripheral Foxp^3+^ regulatory T cells (Tregs) (83). The mechanistic basis for this effect is becoming increasingly clear. HDAC inhibition increases acetylation at the *Foxp3* locus, facilitating its transcription. Concurrently, GPR109A signaling on dendritic cells and macrophages fosters a tolerogenic phenotype that favors Treg differentiation (84). In the lung, this translates into enhanced Treg suppressive capacity, dampened DC maturation, and a shift in alveolar macrophage polarization away from pro-inflammatory states (85). Beyond Tregs, emerging evidence implicates SCFAs in modulating the Th17/neutrophilic axis. For instance, propionate can suppress Th17 differentiation, suggesting that SCFA deficiency might not only exacerbate T2 inflammation but also contribute to non-T2 phenotypes by unleashing Th17-driven neutrophilic responses (86, 87). This has significant implications for understanding the role of the gut-lung axis in neutrophilic and obesity-associated asthma, where metabolic and inflammatory signals are particularly intertwined.

The importance of SCFAs extends well beyond the immediate immune environment. Experimental models demonstrate that maternal or early-life SCFA enrichment reduces offspring susceptibility to allergic airway inflammation. Conversely, early deficits produce durable shifts in hematopoietic progenitor epigenetic landscapes that bias toward exaggerated myelopoiesis (88–90). These findings suggest that SCFA signaling during critical developmental windows may have lasting consequences for asthma susceptibility—a notion with profound implications for primary prevention.

Consistent with these mechanistic insights, patients with asthma exhibit consistently reduced fecal and circulating SCFA levels, particularly in those with severe, eosinophilic phenotypes (20, 74–76). This deficiency correlates with impaired Treg frequencies and increased type 2 cytokine production upon allergen challenge, providing a compelling link between microbial metabolism and disease activity.

#### 3.2.1 The concentration paradox and other unresolved questions

A long-standing puzzle in the field—often referred to as the SCFA concentration paradox—centers on a deceptively simple observation. SCFAs reach millimolar (mM) concentrations in the gut lumen but only low micromolar (μM) levels systemically. This raises questions about whether direct HDAC inhibition can occur in distal organs such as the lung, where concentrations appear far below the typical IC50 values for HDAC inhibition observed *in vitro*. Four non-mutually exclusive hypotheses have been proposed to resolve this paradox. Each generates testable predictions and has distinct implications for therapeutic development. Local education within gut-associated lymphoid tissues (GALT) may occur at mM concentrations due to diffusion gradients from the lumen. This would condition immune cells before they egress into the circulation (91). If this hypothesis holds, GALT Tregs—but not circulating Tregs—should exhibit signatures of HDAC inhibition. This prediction can be tested through single-cell epigenomic profiling of Tregs from different anatomical compartments.

Inflamed lung tissues may exhibit higher local SCFA concentrations than circulating blood. Increased vascular permeability and leukocyte recruitment could create microenvironments where SCFAs accumulate to functionally relevant levels (85). This predicts a correlation between local SCFA levels (measured by microdialysis in asthmatic lungs) and HDAC activity in resident immune cells (85).

Receptor-mediated sensitivity could allow low (nM-μM) SCFA concentrations to exert effects through GPR41 and GPR43. Downstream signaling could potentially amplify HDAC-inhibitory outcomes via secondary messengers (92, 93). A key prediction of this hypothesis is that GPR43/41 knockout should abrogate SCFA effects at low concentrations—a prediction amenable to testing using receptor-deficient mouse models.

Metabolic reprogramming offers an HDAC-independent pathway. SCFAs can enter the tricarboxylic acid (TCA) cycle, shifting immune cell metabolism from glycolysis (pro-inflammatory) toward oxidative phosphorylation (anti-inflammatory) (94, 95). If this mechanism is operative, TCA cycle intermediates such as citrate or succinate should mimic the immunomodulatory effects of SCFAs (96, 97).

Current evidence supports all four mechanisms operating in parallel. However, their relative contributions likely depend on tissue context, inflammatory state, and asthma endotype. Emerging technologies—including spatial metabolomics, advanced *in vivo* microdialysis, and single-cell metabolomic profiling—offer promising avenues to definitively test these hypotheses. These approaches can directly measure SCFA concentrations and receptor occupancy at physiologically relevant sites, potentially resolving the paradox.

Supporting the clinical relevance of these pathways, ex-vivo studies using peripheral blood mononuclear cells from asthmatic adults have shown that treatment with propionate or butyrate directly suppresses Th2 cytokine production (IL-5, IL-13) without requiring prior *in-vivo* conditioning (98). This observation indicates that SCFAs can act directly on differentiated human allergic effector cells, providing a cellular rationale for SCFA-based interventions even in established asthma-not only in early-life prevention.

### 3.3 Bile acids, tryptophan metabolites, and glycan signals

While SCFAs have received the most attention, other microbial metabolites contribute substantially to gut-lung communication. Among these, bile acids provide an archetype of host-microbe co-metabolism with broad immunological consequences.

Primary bile acids undergo bacterial deconjugation and 7α-dehydroxylation in the colon. This microbial processing produces secondary bile acids such as lithocholic acid (LCA) and deoxycholic acid (DCA) (27). These molecules signal through both membrane G-protein coupled receptors (TGR5) and nuclear receptors (FXR). Some secondary bile acids or their derivatives also engage the aryl hydrocarbon receptor (AhR). TGR5 activation elevates intracellular cAMP in macrophages and dendritic cells, promoting anti-inflammatory phenotypes and suppressing NF-κB–driven cytokine production (77). FXR signaling influences epithelial barrier integrity and suppresses Th17 polarization (99). Disturbances in bile acid profiles—arising from dysbiosis, altered hepatic function, or diet—can therefore either protect against or predispose to airway inflammation.

The relationship between bile acids and asthma is bidirectional. Asthma-associated inflammation can alter hepatic bile acid synthesis, while gut dysbiosis can shift the bile acid pool toward pro-inflammatory species. This complex interplay highlights the need for longitudinal studies that capture dynamic changes in bile acid metabolism across disease trajectories.

Tryptophan metabolism represents another critical node connecting gut microbial ecology to pulmonary immunity. Tryptophan is metabolized along parallel host- and microbe-driven pathways (28). Host indoleamine-2,3-dioxygenase (IDO) channels tryptophan toward the kynurenine pathway (100). In contrast, gut bacteria—notably *Lactobacillus* and certain *Clostridia*—convert tryptophan into indole derivatives such as indole-3-aldehyde (I3A) and indole-3-propionic acid (IPA) that act as potent AhR agonists (29).

AhR signaling is critical for multiple aspects of mucosal homeostasis. It enhances epithelial barrier function, promotes IL-22 production from ILC3s, and helps balance Treg/Th17 differentiation (101). In pulmonary contexts, AhR activation shapes alveolar macrophage function and can restrain pathogenic type 2 responses. The balance between IDO-driven kynurenines and microbially produced indoles may be a decisive determinant of asthma endotypes. Patients with allergic asthma often show reduced circulating levels of IPA and I3A, suggesting that diminished microbial tryptophan metabolism contributes to disease pathogenesis (28).

Complex glycans add another layer of metabolic complexity. Polysaccharide A (PSA) from Bacteroides fragilis act through TLR2 to induce IL-10 production and Treg expansion (102–104). Beyond freely diffusible small molecules, microbes secrete extracellular vehicles (EVs) that encapsulate lipids, proteins, and nucleic acids (79, 105, 106). These EVs can cross epithelial barriers, enter the circulation, and deliver concentrated molecular cargos to distant sites, including the lung (106, 107). The emerging recognition of EV-mediated communication adds yet another dimension to the gut-lung axis and suggests novel therapeutic opportunities.

### 3.4 Remote immune cell education and trafficking

Metabolites alone do not tell the full story of gut-lung communication. The gut mucosa is also a major inductive site for immune education (108). Through cellular sampling of luminal contents, antigen presentation within gut-associated lymphoid tissues, and metabolite-driven conditioning, the intestine shapes the phenotype, trafficking capacity, and functional set-points of multiple immune lineages (109–111). These gut-educated cells can migrate systemically and seed the lung, acting as cellular conduits that carry ecological memory from the gut to the respiratory mucosa (112).

#### 3.4.1 Regulatory T cells: from colonic induction to pulmonary suppression

Microbially induced Foxp3^+^ Tregs represent a paradigmatic mechanism by which gut ecology imposes systemic immune tolerance (113). Clostridial clusters IV and XIVa, via butyrate production and other signals, promote peripheral Treg differentiation through HDAC inhibition and retinoic acid–dependent pathways (114). Tregs induced in the colonic environment express gut-homing receptors such as CCR9 and integrin α4β7. However, a subset acquires broader trafficking phenotypes. These cells express receptors and adhesion molecules that permit migration to extra-intestinal mucosae including the lung (115).

Once in the lung, these Tregs suppress allergen-driven effector T-cell responses. They limit eosinophil recruitment and restrain type 2 cytokine production (116–118). Their abundance and functional competency correlate with reduced allergic sensitization in murine models. They are emerging as candidate biomarkers of resilient mucosal immunity in humans (119, 120). In asthma, the frequency and suppressive function of circulating and airway Tregs are diminished, particularly during exacerbations. This deficiency has been linked to reduced abundance of butyrate-producing bacteria (115). Preclinically, restoring Treg numbers through probiotic interventions or butyrate supplementation attenuates airway hyperresponsiveness in murine models. These findings suggest that the gut-Treg-lung axis represents a promising target for disease-modifying interventions.

#### 3.4.2 Innate lymphoid cells: gut imprinting and distant effector mobilization

Innate lymphoid cells, particularly ILC2s and ILC3s, exemplify how innate populations can be shaped in the gut and subsequently influence pulmonary outcomes (121). Gut stimuli—including epithelial alarmins (IL-25, IL-33), lipid mediators, and microbial metabolites—modulate ILC phenotype, proliferation, and expression of homing receptors (122). The induction of S1PR1 on ILC2s following protozoan or other microbial stimuli enables egress from gut tissues and homing to the lung along sphingosine-1-phosphate gradients. In the lung, these cells act as rapid sources of IL-5 and IL-13 upon allergen exposure (123, 124). Conversely, microbial signals favoring ILC3 and IL-22 production reinforce barrier function and may mitigate allergic priming (125). The balance between ILC2 and ILC3 responses in the gut may thus determine the trajectory of allergic sensitization in the lung.

#### 3.4.3 Dendritic cells and tolerogenic imprinting

Intestinal dendritic cells continually sample luminal antigens and integrate microbial cues. They assume tolerogenic or immunogenic phenotypes based on the signals received. Microbial metabolites (SCFAs, bile acids, PSA) and epithelial signals (retinoic acid) condition DCs to produce IL-10, TGF-β, and retinaldehyde dehydrogenase. The latter catalyzes retinoic acid production, which imprints gut-homing phenotypes on T cells (119, 120). Tolerogenic DCs can migrate to mesenteric lymph nodes to induce Tregs. Soluble mediators or migrating DC subsets may carry tolerogenic signals systemically (126). In the lung, the presence of DCs with gut-imprinted tolerogenic signatures correlates with attenuated allergen presentation and reduced Th2 priming (127). This finding underscores the importance of the gut-DC-Treg axis in maintaining pulmonary tolerance.

### 3.5 Mechanistic convergence: receptors, epigenetics, and metabolic state

The pathways described above converge on several core principles that transcend individual metabolites or cell types. Microbial metabolites act through specific receptors—GPCRs, nuclear receptors—that translate chemical signals into cellular responses.This receptor-mediated signaling is remarkably pleiotropic, with the same metabolite activating different pathways in different cell types depending on receptor expression patterns.

Metabolites like butyrate function as HDAC inhibitors, inducing epigenetic changes that can persist long after the metabolite has been cleared. This provides a mechanism for durable immunological imprinting. The epigenetic effects of SCFAs on hematopoietic progenitors, for example, may explain why early-life microbial exposures have lasting consequences for asthma susceptibility. Recent work has extended this epigenetic paradigm to include RNA modifications. A groundbreaking study by Zhang et al. demonstrated that host m6A modifications can shape the gut microbiota, which in turn drives cell-specific ferroptosis as a causal pathway to chronic respiratory diseases. This work introduces two novel elements—RNA methylation and ferroptosis—into the gut-lung axis framework. Ferroptosis, an iron-dependent form of regulated cell death, had not previously been linked to microbiome-mediated lung inflammation. The study by Zhang et al. suggests that specific microbial communities, influenced by host epigenetic modifications, can trigger ferroptosis in lung cells, thereby contributing to disease pathogenesis. This represents an exciting new frontier that expands our mechanistic understanding beyond traditional immune paradigms and underscores the importance of including cell death pathways in future studies (128). Metabolites can enter central carbon metabolism, shifting energy production from glycolysis to oxidative phosphorylation. This metabolic reprogramming is associated with anti-inflammatory phenotypes across multiple immune lineages (129). Finally, signals act at multiple levels—local (gut epithelium), regional (GALT), systemic (circulation), and distal (bone marrow, lung)—creating a layered regulatory network. This multi-level architecture provides redundancy and resilience but also complicates therapeutic targeting.

### 3.6 Timing, developmental windows, and experimental tools

Perinatal and early-life windows are periods of heightened plasticity during which microbial signals can set durable immune trajectories. These signals shape hematopoietic stem and progenitor cells (HSPCs), establish tissue-resident immune repertoires, and license epithelial barrier maturation (130). Interventions during these windows have the potential to prevent allergic sensitization. Interventions in established disease may require combination approaches to reset epigenetic programs (131).

Causal inference in this arena relies on multiple complementary approaches. Germ-free and gnotobiotic models allow controlled dissection of microbe-host interactions. Targeted microbial consortia enable testing of specific community configurations. Stable isotope tracing tracks metabolic flux from gut to periphery. Single-cell transcriptomics reveals cell-type-specific responses. Fate-mapping tracks cell migration and differentiation. Parabiosis establishes whether signals are blood-borne. Mendelian randomization tests causal relationships at the population level (55). Spatial transcriptomics and imaging mass spectrometry are enabling localization of metabolites and receptors at mucosal interfaces. The integration of these approaches promises to accelerate the transition from correlation to causation in gut-lung axis research.

### 3.7 Asthma endotype-specific considerations

The preceding sections have largely focused on the T2-high eosinophilic asthma paradigm, given its strong and reproducible association with gut microbial metabolites like SCFAs. However, the role of the gut-lung axis in non-T2 asthma remains an under-explored frontier that warrants specific attention. Neutrophilic asthma, often associated with obesity and corticosteroid resistance, is characterized by Th17-driven inflammation. Preclinical studies suggest that gut dysbiosis and reduced SCFAs can promote Th17 differentiation, potentially linking microbial dysfunction to this difficult-to-treat phenotype (132, 133). Furthermore, the gut microbiota’s influence on systemic metabolism, particularly through bile acids and other metabolites, may be pivotal in obesity-associated asthma, where both chronic low-grade inflammation and altered microbial ecology are prominent (134, 135). Paucigranulocytic asthma, which lacks significant eosinophil or neutrophil infiltration, might be linked to a distinct set of microbial or metabolic perturbations, possibly involving impaired Treg function without the induction of classic effector responses (136, 137). Future research should prioritize characterizing the gut-lung axis in these understudied endotypes to determine if shared or distinct mechanistic pathways exist, which is critical for the development of truly personalized microbiome-directed therapies.

### 3.8 The reverse route: pulmonary signals shaping the gut microbiome

The primary focus of this review is gut-to-lung communication. However, the reciprocal influence of pulmonary inflammation and infection on the gut microbiome merits explicit consideration, as it completes the conceptual framework of a truly bidirectional axis. The lung-to-gut pathway remains mechanistically less defined but appears to operate through several interconnected mechanisms. First, systemic inflammation triggered by airway disease—notably the release of cytokines such as IL-6 and TNF-α—can directly impact the intestinal epithelium, compromising gut barrier integrity and reshaping the local microbial niche (138). This inflammatory milieu may favor the expansion of pathobionts within the gut lumen. Second, pharmacological interventions commonly used to manage asthma, including antibiotics and corticosteroids, exert profound and selective pressures on the gut microbiota; this drug-induced modulation represents an indirect yet clinically significant form of lung-to-gut signaling (139). Third, emerging evidence implicates neuro-immune pathways, particularly vagus nerve-mediated signaling, in transmitting inflammatory signals from the lung to the gut, thereby linking the two organs through a neural reflex arc (140). Finally, systemic alterations in metabolism and oxygen tension during respiratory distress may further influence gut microbial composition (141). This reverse pathway poses a substantial confounding factor in observational studies: it remains challenging to discern whether observed gut dysbiosis represents a cause or a consequence of airway inflammation. Dedicated longitudinal studies capable of capturing simultaneous pulmonary and intestinal inflammation are urgently needed to disentangle these complex dynamics and to rigorously establish the clinical bidirectionality of this axis.

## 4 From association to causation: integrating multi-omics and causal inference

The mechanistic pathways described in Sections 2 and 3 provide a rich landscape of testable hypotheses linking specific microbial functions to asthma phenotypes. However, a fundamental challenge remains: moving from compelling mechanistic insights to actionable clinical targets requires rigorous causal inference. Traditional association studies cannot distinguish cause from consequence, but recent advances in multi-omics integration, Mendelian randomization, and experimental triangulation are transforming the field from associative cartography to a mechanistic discipline.

This section addresses a question that has long plagued microbiome research: which microbial features are truly causal drivers of asthma pathogenesis, and which are merely bystanders altered by disease processes or confounding factors? The distinction has profound implications for therapeutic development, biomarker discovery, and our fundamental understanding of disease mechanisms.

### 4.1 Design principles for multi-omics studies

Robust causal inference begins with well-designed multi-omics studies. A thoughtfully constructed study incorporates longitudinal sampling across critical developmental windows—prenatal, infancy, and peri-exacerbation periods. This temporal dimension is essential for establishing whether microbial changes precede disease onset or follow it. Deep clinical phenotyping captures allergen sensitization and environmental exposures, medication use, and disease severity with sufficient granularity to enable meaningful stratification. Multi-compartment sampling from stool, blood, and airway specimens captures the full spectrum of microbial-host interactions. Uniform biobanking protocols minimize batch effects that could obscure true biological signals.

With such designs in place, an integrated pipeline can be deployed. Shotgun metagenomics defines taxonomic composition and functional potential, while metatranscriptomics reveals community gene expression. Metaproteomics and metabolomics provide the proximate readout of effector molecules, and host transcriptomics, epigenomics, and high-dimensional immune phenotyping map the consequent immune states. Emerging spatial omics technologies further localize these interactions within native tissue architecture.

A critical insight emerging from these studies is that taxonomic shifts are often context-dependent. A given bacterial species may be protective in one population and pathogenic in another, depending on the broader community context, host genetics, and environmental exposures. Function-centric approaches that focus on metabolic pathway capacities and metabolite levels are therefore more portable and mechanistically informative than taxonomic summaries alone. Tools such as HUMAnN and genome-scale metabolic models enable mapping from sequencing data to community metabolic potential, providing a functional lens through which to interpret community composition.

Dimension reduction methods like Multi-Omics Factor Analysis (MOFA) can capture latent factors representing biologically coherent modules – for example, a butyrate synthesis module linked to serum butyrate and Treg signatures. Network and causal graph approaches reveal modules of co-regulated taxa and genes, suggesting functional relationships that can be tested experimentally. Causal discovery algorithms, such as those based on directed acyclic graphs or structural equation modeling, generate directional hypotheses that require corroboration with orthogonal methods. These computational approaches are not substitutes for experimentation but rather hypothesis-generating tools that prioritize the most promising relationships for mechanistic investigation.

### 4.2 Mendelian randomization and genetic inference

Mendelian randomization (MR) offers a powerful genetic approach to infer causality by exploiting the random allocation of alleles at conception (142, 143). Genetic variants robustly associated with an exposure—microbial abundance or metabolite level, for example—serve as instrumental variables to test for causal effects on asthma outcomes. Because alleles are randomly assorted at meiosis, MR is less susceptible to confounding and reverse causation than observational studies. This makes it a valuable tool for prioritizing causal relationships for further investigation.

The validity of MR depends on three core assumptions, each of which faces distinct challenges in microbiome research. The first assumption is that the instrumental variables are robustly associated with the exposure. This is often challenging in microbiome MR because microbial quantitative trait loci (mbQTLs) typically explain only a small fraction of variance in microbial traits. Weak instruments can lead to biased estimates and inflated false-positive rates. Aggregating variants into polygenic scores may improve instrument strength but risks introducing pleiotropy, violating the second assumption.

The second assumption is that the instrumental variables affect the outcome only through the exposure of interest—that is, there is no horizontal pleiotropy. This is particularly challenging in microbiome research because genetic variants associated with one microbial trait may also affect host immunity, diet, or other factors that independently influence asthma risk. Horizontal pleiotropy can be detected and corrected using methods like MR-Egger regression, which tests whether the intercept deviates from zero, indicating pleiotropic effects. However, these methods have limited power and may not detect all forms of pleiotropy.

The third assumption is that there is no population stratification—that is, the associations are not confounded by ancestry. This necessitates careful matching or family-based designs. Population stratification is a particular concern in microbiome research because both gut microbiota composition and asthma risk vary substantially across ancestral backgrounds.

Advanced MR frameworks, including multivariable and mediation MR, enable testing of causal chains. For example, multivariable MR can assess whether the effect of a microbial trait on asthma is mediated by a specific metabolite or immune cell type. Mediation MR can quantify the proportion of the total effect that operates through a proposed intermediate. These approaches are still in their infancy in microbiome research but hold considerable promise. A recent study by Bi et al. provides an empirical illustration of this approach, demonstrating a causal relationship between the gut microbiota and asthma, with blood cell perturbations acting as a significant mediating variable. Their work not only reinforces the potential of MR for causal discovery but also explicitly addresses the reverse-causation concern by showing that the mediation effect is robust to bidirectional testing (144).

Beyond genetic inference, modern causal machine learning approaches preserve statistical robustness while handling high-dimensional microbiome data. Targeted maximum likelihood estimation (TMLE) and doubly robust estimators allow flexible models for high-dimensional microbiome predictors while maintaining unbiasedness. These methods are less sensitive to model misspecification than traditional regression approaches. Causal forests and uplift modeling can estimate treatment effect heterogeneity, identifying which patients would most benefit from a given microbiome-targeted intervention. This is particularly valuable for designing biomarker-enriched clinical trials.

### 4.3 The butyrate–Treg–asthma causal chain: a worked example

To illustrate the practical application of the proposed causal inference framework, we examine the most extensively investigated candidate pathway in the gut-lung axis: reduced microbial butyrate production leading to impaired regulatory T cell differentiation and consequent eosinophilic airway inflammation. This pathway serves as an instructive paradigm because it has been interrogated through multiple lines of evidence—associational, genetic, temporal, mechanistic, and interventional—allowing assessment of both the strength and the limitations of current causal claims.

#### 4.3.1 Associational evidence

The associational evidence for this pathway is robust. Meta-analyses of case—control studies have consistently demonstrated reduced fecal abundance of butyrate-producing taxa, particularly Faecalibacterium prausnitzii, as well as decreased circulating SCFA levels, in patients with eosinophilic asthma compared with healthy controls (76, 91, 98). While these associations are consistent across multiple cohorts, they do not by themselves establish causation. Reverse causality—whereby asthma-associated inflammation alters gut microbial composition—remains a plausible alternative. Confounding by diet, medication use, or other factors could also explain the observed associations (129, 145, 146).

Genetic Evidence. Genetic approaches have been employed to address the limitations of observational studies. However, the genetic evidence for a causal relationship between butyrate production and asthma specifically is more limited than for metabolic diseases. Early MR studies using mbQTLs as instrumental variables for butyrate production capacity provided suggestive evidence for a causal effect on metabolic disease outcomes. For asthma, more recent work has begun to address this gap, including studies on host genetics and gut microbiota in US Hispanic/Latino adults (147) and large-scale consortium analyses of host genetic influences on gut microbiome composition (143). However, the proportion of variance in butyrate production explained by currently identified mbQTLs remains modest, and the MR-Egger intercept test often suggests potential pleiotropy. These caveats underscore the need for larger genome-wide association studies with improved power to detect mbQTLs and for multivariable MR approaches that can account for potential confounding by correlated microbial traits.

#### 4.3.2 Temporal evidence

Temporal precedence, a core criterion for causation, is supported by longitudinal birth cohort studies. Depner et al. demonstrated that maturation of the gut microbiome during the first year of life contributes to the protective farm effect on childhood asthma (31). This temporal relationship strengthens the case for a causal direction from early-life microbial alterations to asthma development. However, it does not exclude the possibility that early butyrate deficiency is a marker of broader environmental or genetic factors that independently influence asthma risk. The challenge of distinguishing markers from mediators is a recurring theme in microbiome epidemiology.

#### 4.3.3 Mechanistic evidence

The most direct mechanistic evidence derives from gnotobiotic mouse models. Mice colonized with a butyrate-deficient microbial consortium exhibit exaggerated airway eosinophilia and type 2 cytokine production following allergen challenge, compared with mice colonized with butyrate-producing consortia (90). Critically, this phenotype is rescued by oral butyrate supplementation, demonstrating that the microbial metabolite itself—rather than other microbial community features—is sufficient to modulate the airway inflammatory response. These experiments establish biological plausibility and define a concrete molecular mechanism involving HDAC inhibition, Foxp3 acetylation, and subsequent Treg differentiation.

Nevertheless, translation of these findings to human asthma requires caution. The controlled conditions of gnotobiotic models—including defined diets, standardized genetic backgrounds, and specific-pathogen-free facilities—do not fully recapitulate the complexity of human microbial ecology, dietary variation, or genetic heterogeneity. Moreover, the dose and timing of butyrate supplementation in mouse models may not correspond to what is achievable or desirable in humans. These limitations highlight the need for complementary approaches that bridge the gap between mechanistic models and human disease.

#### 4.3.4 Intervention evidence in humans

Direct interventional evidence for butyrate in human asthma remains limited. To date, only one exploratory pilot trial has evaluated a butyrate-containing immunonutritional supplement in children with allergic asthma (INAPRA study) (148). While this study provided preliminary feasibility data, it was limited by its small sample size, complex multi-component formulation, and lack of significant effects on objective physiological endpoints such as FEV1 and FeNO. These inconclusive results underscore the urgent need for larger, adequately powered phase 2 randomized controlled trials that are stratified by baseline SCFA deficiency and incorporate mechanistic endpoints to confirm target engagement. Until such trials are conducted, the clinical efficacy of butyrate supplementation in asthma remains unproven.

#### 4.3.5 Synthesis and next steps

In summary, the butyrate-Treg-asthma causal chain is supported by convergent evidence from observational epidemiology, genetic inference, temporal cohort studies, mechanistic animal models, and preliminary human intervention data. Nevertheless, definitive proof of causation in humans remains elusive. The next critical steps should include larger Mendelian randomization studies with more powerful instruments for butyrate production, ideally using multi-tissue and multi-ancestry approaches. Phase 2 randomized controlled trials stratified by baseline butyrate status are needed to test whether supplementation provides clinical benefit specifically in SCFA-deficient asthmatics. Integrated multi-omics analyses that combine metagenomic, metabolomic, and host transcriptomic data to identify effect modifiers and predictive biomarkers of response. This framework, illustrated here for the butyrate pathway, can be systematically applied to other candidate mediators—including bile acids, tryptophan metabolites, and fungal or protozoan signals—to prioritize the most promising targets for therapeutic development.

### 4.4 Experimental triangulation and validation platforms

No single method is sufficient to establish causality. Instead, experimental triangulation combines multiple lines of evidence to build confidence. A robust causal chain integrates genetic causality (MR using mbQTLs), temporal precedence (longitudinal cohorts showing exposure precedes outcome), mechanistic sufficiency (gnotobiotic models, purified metabolite administration), and intervention evidence (randomized controlled trials with biomarker-based enrichment). When multiple approaches converge on the same causal relationship, confidence increases substantially. When they diverge, the discrepancies often reveal important nuances—for example, that an effect is context-dependent or mediated through unexpected pathways.

Several experimental platforms are particularly valuable for causal dissection. Humanized gnotobiotic mice colonized with defined human microbial consortia allow controlled testing of specific community configurations without the confounding variables present in human studies. Intestinal and airway organoids enable mechanistic studies of epithelial–microbe interactions in a controlled, human-relevant context. Ex vivo tissue explants and precision-cut lung slices from human lung resections provide a bridge between reductionist models and whole-organ physiology. Microphysiological systems (multi-organ chips) that integrate gut, liver, and lung compartments with vascular flow enable dynamic studies of metabolite trafficking and inter-organ communication. These platforms are particularly valuable for dissecting species-specific mechanisms that may not be captured in standard mouse models.

The choice of platform depends on the specific question being asked. For studies of microbial metabolites, organoid and microphysiological systems offer the advantage of human cells while maintaining experimental control. For studies of immune cell trafficking, gnotobiotic mice with defined microbial communities enable lineage tracing and functional interrogation. For translational studies, humanized models and ex vivo systems provide the most direct relevance to human disease. A pragmatic approach combines platforms in a staged manner, starting with high-throughput screening in simpler systems and progressing to more complex models for mechanistic validation.

### 4.5 Proposed roadmap for causal discovery

To move the field forward, we propose a staged roadmap that emphasizes convergence rather than reliance on any single approach. The framework unfolds across three interconnected phases. The discovery phase begins with evaluating candidate causal relationships for biological plausibility, consistency across cohorts, and therapeutic potential; those supported by multiple lines of evidence—consistent associations, temporal precedence, and plausible mechanisms—should be prioritized for deeper investigation, with genetic triangulation using Mendelian randomization and mbQTLs from diverse populations offering a population-level test of causal hypotheses. The mechanistic validation phase shifts to controlled experimental settings, where gnotobiotic models, defined microbial consortia, and purified metabolite experiments can determine whether candidate relationships are truly causal; these studies should assess dose-response relationships, timing of intervention, and cell-type specificity while identifying the molecular pathways through which microbial signals influence host physiology. The intervention phase represents the ultimate test of clinical utility: biomarker-enriched randomized controlled trials targeting specific microbial functions—butyrate supplementation in SCFA-deficient asthmatics, for instance—provide the most direct evidence of therapeutic benefit, with mechanistic endpoints to confirm target engagement and distinguish responders from non-responders, and adaptive designs to accelerate identification of effective interventions while minimizing exposure to ineffective ones. This roadmap is not a rigid prescription but a flexible framework adaptable to specific questions and resources; the guiding principle is convergence—when multiple lines of evidence point in the same direction, confidence grows, and when they diverge, the discrepancies often reveal important nuances that advance our understanding.

## 5 Translational frontiers: towards microbiome-informed precision medicine

With robust causal frameworks in place, the field is now poised to translate mechanistic insights into clinical applications. This section critically examines the emerging pipeline of microbiome-informed diagnostics and therapeutics. Rather than cataloguing every potential approach, we focus on interventions that have reached clinical testing or are supported by strong preclinical evidence, and we explicitly distinguish between what is established, what is promising, and what remains speculative (**Figure 1**).

**Figure 1:**
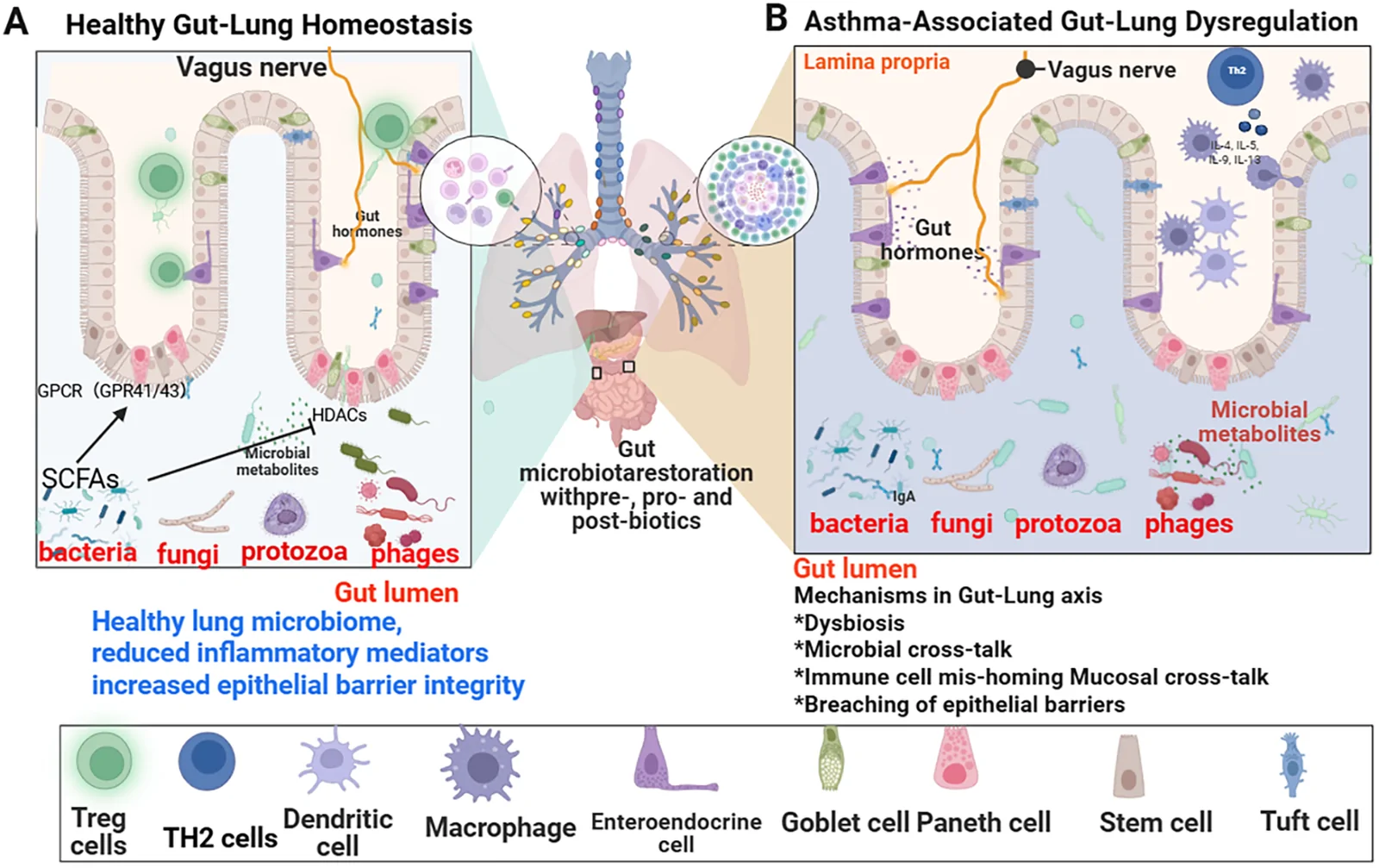
The gut–lung axis in health and asthma. This schematic contrasts gut–lung axis homeostasis [**(A)**, healthy state] with dysregulation in asthma **(B)**. In health, a diverse gut microbiota produces metabolites such as short-chain fatty acids (SCFAs). These signal via GPCRs (GPR41/43) and inhibit histone deacetylases (HDACs). This promotes regulatory T cell (Treg) differentiation and epithelial barrier integrity. The vagus nerve and gut hormones further mediate gut–lung communication, supporting a healthy lung microbiome, reduced inflammation, and intact airway epithelium. In asthma **(B)**, gut dysbiosis leading to altered microbial metabolites, impaired barrier function, and increased type 2 inflammation. Th2 cells producing IL-4/IL-5/IL-9/IL-13 drive pro-inflammatory responses, while disrupted vagal signalling and gut hormone release contribute to immune miscommunication between the gut and lung.

A recurring theme throughout this section is the importance of patient stratification. The heterogeneity of asthma—encompassing multiple endotypes, varying severity, and diverse environmental triggers—means that a single microbial therapeutic strategy is unlikely to benefit all patients. Biomarker-based enrichment, guided by the diagnostic and stratification tools discussed below, will be essential for demonstrating clinical utility in future trials.

### 5.1 Diagnostic and stratification biomarkers

The development of reliable biomarkers is a prerequisite for precision medicine in asthma. Several candidate biomarker classes have emerged from microbiome research, each with distinct advantages and limitations (**Table 3**).

**Table 3:** Candidate biomarkers for microbiome-informed asthma stratification and clinical utility.

Biomarker category	Specific marker	Association with asthma	Potential clinical utility	Sample source	Clinical accessibility	References
Microbial signature	*Faecalibacterium prausnitzii* abundance	Decreased in eosinophilic asthma; inversely correlates with exacerbation frequency across multiple cohorts	Diagnostic; predictor of SCFA deficiency; risk stratification	Feces	Research-only	(24, 152)
Microbial signature	Butyrate production capacity gene cluster (butyryl-CoA: acetate CoA-transferase)	Reduced metagenomic capacity for butyrate synthesis in severe, steroid-refractory asthma vs. mild/moderate	Prospective assessment of functional deficit could guide prebiotic/probiotic selection	Feces (metagenomic)	Research-only	(25, 26)
Microbial ratio	Bacteroides/Lactobacillus ratio (relative abundance)	Positive correlation with disease severity; elevated in corticosteroid-exposed asthmatics	Prognostic stratification; monitoring of microbiome disruption from steroid/antibiotic therapy	Feces	Research-only	(153, 154)
Metabolite	Fecal/circulating SCFAs (sum butyrate + propionate + acetate)	Reduced (40-50%) in severe asthma compared to mild; correlates with lower Treg frequencies (peripheral blood)	Monitor inflammatory status; guide prebiotic/postbiotic therapy	Feces, serum	Emerging (specialized centers)	(20, 74, 75)
Metabolite	Tryptophan metabolites (indole-3-propionic acid, indole-3-aldehyde)	Decreased IPA/I3A in allergic asthma vs. healthy controls; inversely correlated with FeNO and blood eosinophils	Predict AhR signaling capacity; monitor of intestinal epithelial barrier function	Serum, feces	Research-only	(28, 29)
Translocation signature	Serum (1→3)-β-D-glucan (fungal cell wall component)	Elevated in severe asthma with fungal sensitization; correlates with type 2 biomarkers (periostin, FeNO) in subsets of patients with fungal translocation	Identify patients with altered gut barrier function; guide antifungal or barrier-restorative therapy	Serum	Emerging (specialized centers)	(71, 155)
Immune-metabolic composite	Treg frequency (peripheral blood Foxp3+ CD45RA+CCR7-) + serum butyrate level	Combined low levels predict exacerbation risk and need for stepped care in severe eosinophilic asthma	Prognostic of exacerbation risk; guide combination (e.g., postbiotic + standard/biological) therapy	Blood + serum	Research-only	(76, 156)

Functional metabolomic profiles reflect proximal microbial activity and can be measured in serum or feces. Circulating SCFAs, bile acids, and tryptophan metabolites provide a readout of community metabolic function that is more directly relevant to host physiology than taxonomic composition alone. These metabolites can be quantified using targeted mass spectrometry, which is increasingly accessible in specialized clinical laboratories. However, standardization across laboratories remains a challenge, and reference ranges for healthy and asthmatic populations are not yet well established.

Microbiome risk scores (MRS) aggregate taxonomic or functional features into a single index. Early versions have shown moderate predictive value for asthma development in birth cohorts (149, 150). The advantage of MRS is their simplicity—a single number summarizes complex microbial data. The disadvantage is that they obscure the specific mechanisms through which microbes influence disease, limiting their utility for guiding targeted interventions.

Integrated host–microbe classifiers combine microbial features with clinical parameters (FeNO, blood eosinophils, lung function). These multimodal approaches may improve diagnostic accuracy beyond either domain alone. For example, combining Faecalibacterium abundance with FeNO and blood eosinophil count may better predict exacerbation risk than any single marker. However, the optimal combination of features and the appropriate weighting of each component remain areas of active investigation.

Translocation signatures, including circulating β-glucan and microbial cell-free DNA, could identify patients with gut barrier dysfunction and fungal translocation (147, 151). These markers are particularly relevant for patients with severe asthma who may have impaired intestinal barrier function due to chronic inflammation or corticosteroid use. However, the specificity of these markers for gut-derived translocation versus other sources of microbial products remains to be established.

Functional immune assays that measure ex vivo responses of patient peripheral blood mononuclear cells to microbial metabolites might capture individual immune set-points. For example, measuring Treg induction in response to butyrate could identify patients who are most likely to benefit from butyrate supplementation. These assays are currently research tools but could be developed into clinical diagnostics.

Development of such biomarkers should follow a staged pathway: discover (identifying candidate markers in discovery cohorts), technical validation (developing robust assays), clinical validation (confirming performance in independent cohorts), and demonstration of clinical utility (showing that biomarker-guided management improves outcomes). This pathway is well established in other areas of medicine and should be applied rigorously to microbiome biomarkers (98).

It is important to distinguish between biomarkers that are currently accessible in standard clinical settings and those that remain research tools. While blood eosinophil counts and FeNO are routinely available, most microbial and metabolomic biomarkers—including metagenomic profiling, fecal SCFA quantification, and serum tryptophan metabolite measurement—remain confined to research laboratories. The Bacteroides/Lactobacillus ratio and serum (1,3)-β-D-glucan assays are available in some specialized centers but lack standardized clinical protocols. Widespread clinical adoption will require assay standardization, cost reduction, and prospective validation of clinical utility.

### 5.2 Targeted intervention strategies

The therapeutic toolbox spans ecological, molecular, cellular, and engineered approaches. Each has distinct advantages and challenges, and levels of evidence. **Table 4** summarizes emerging microbiome-targeted interventions for asthma, stratified by evidence strength.

**Table 4:** Emerging microbiome-targeted interventions for asthma: evidence and mechanisms.

Intervention category	Specific agent/strategy	Evidence level*	Key findings	Next steps	References
Precision nutrition/prebiotics	High-fiber diet; inulin; resistant starch	Moderate (Phase 2 pilot)	High inter-individual variability; no FEV1 improvement	Endotype-stratified RCT	(157, 158)
Probiotics/synbiotics	Lactobacillus spp., Bifidobacterium spp.	Moderate (Meta-analysis, 2024, n=12 RCTs, N = 1,401)	No objective physiological improvement; high strain heterogeneity	Strain-specific, endotype-stratified trials	(159, 160)
Postbiotics	Strain-specific, endotype-stratified trials SCFA supplements (butyrate, tributyrin); heat-killed Faecalibacterium	Low (Phase 1 pilot)	Small sample; no clinical endpoints; requires formulation optimization	Phase 2 RCT with biomarker enrichment	(74, 75)
Fecal microbiota transplantation (FMT)	Healthy donor FMT	Very Low (Case reports)	No human efficacy data; safety concerns	Not recommended outside research; RCTs required	(161, 162)
Engineered live biotherapeutics (eLBPs)	IL-10-producing Lactococcus lactis	Theoretical (Preclinical in other diseases)	No asthma-specific data; regulatory hurdles	Preclinical asthma models	(163, 164);
Phage therapy	Caudovirales cocktails targeting Enterobacteriaceae	Theoretical (Preclinical in other applications)	No asthma-specific data; phage resistance	Preclinical proof-of-concept	(34, 165)

Evidence Level Definitions: High = Multiple RCTs or meta-analysis with consistent findings; Moderate = Meta-analysis with limitations, or consistent observational data; Low = Single pilot study or inconsistent evidence; Very Low = Case reports or animal data only; Theoretical = Conceptually promising but no direct evidence.

Precision nutrition and prebiotics aim to match fermentable substrates to an individual’s baseline microbiome capacity. Patients with low *Faecalibacterium* abundance may benefit from inulin or resistant starch, whereas those with intact SCFA-producing capacity might not respond. Several small trials have shown that high-fiber diets increase fecal SCFAs and modestly improve asthma control, but responses vary considerably (157, 158, 166). The variability in response likely reflects differences in baseline microbiome composition, dietary patterns, and host genetics. Future studies should incorporate baseline microbiome profiling to identify responders and non-responders, moving beyond the “one-size-fits-all” approach that has limited the impact of dietary interventions.

Probiotics have been extensively studied in asthma, with mixed results. A 2024 systematic review and meta-analysis of 12 randomised controlled trials (1,401 participants) found that probiotics significantly improved asthma control test scores (OR 1.18, 95% CI 1.18–3.64). However, no significant effects were detected on FeNO or blood eosinophil levels (160). These findings suggest that current probiotic interventions primarily affect symptom control rather than objective physiological markers. The heterogeneity in probiotic strains, doses, and patient populations complicates interpretation. Strain-specific effects are likely—for example, certain Lactobacillus strains may be more effective than others—but head-to-head comparisons are lacking. Future trials should be stratified by baseline microbiome characteristics and should incorporate mechanistic endpoints to identify the active principles.

Postbiotics—preparations of inanimate microorganisms or their metabolites—offer several advantages over live probiotics. They provide controlled dosing, simpler regulatory pathways, and no risk of live microbial translocation. Preliminary human data have begun to emerge. An exploratory pilot randomized trial (n=40) evaluated a multicomponent immunonutritional supplement containing sodium butyrate in children with allergic asthma and rhinitis (INAPRA study). While this study employed a complex supplementation strategy rather than butyrate alone, it represents an important step toward clinical translation. However, the lack of significant effects on objective physiological parameters underscores the need for larger, adequately powered trials with biomarker-based patient enrichment.

Fecal microbiota transplantation (FMT) remains exploratory for severe, refractory asthma. In a rat model of ovalbumin-induced allergic asthma, FMT from healthy donors restored gut SCFA levels and attenuated airway inflammation (161). In humans, only case reports exist to date, and FMT is not recommended outside research settings (167). The risks of FMT—including infection transmission, dysbiosis, and unknown long-term effects—must be weighed against potential benefits. If FMT is to be pursued for asthma, it should be restricted to carefully selected patients in the context of well-designed clinical trials with rigorous safety monitoring.

Engineered live biotherapeutic products (eLBPs) represent a frontier in microbiome therapeutics. These “smart therapeutics” incorporate sensor-actuator circuits and built-in safety switches. For example, Lactococcus lactis engineered to produce IL-10 has shown efficacy in preclinical colitis models (163). No eLBPs have yet been tested in asthma, but the concept is promising.

Several challenges currently limit clinical translation. Biological containment remains a primary concern: engineered organisms must be equipped with biocontainment systems—auxotrophic dependencies, kill switches, or physical encapsulation—to prevent environmental persistence and horizontal gene transfer of synthetic circuits to native gut microbes (163, 164). Regulatory pathways for live engineered organisms are still evolving; no eLBP has yet been approved for chronic inflammatory diseases. The FDA has established a framework for live biotherapeutic products, but specific guidance for genetically engineered strains remains under development. Finally, the complex ecological dynamics of the gut microbiome—competition with native strains, phage predation, and nutrient limitations—may prevent stable engraftment of introduced eLBPs. This may necessitate repeated dosing or combination with prebiotic substrates.

Phage therapy offers precision modulation of bacterial populations. Bacteriophages targeting specific pro-inflammatory pathobionts could reduce their abundance without broad dysbiosis. No asthma-specific phage data exist, but the approach is theoretically sound and merits preclinical exploration Key challenges include phage resistance, delivery to the gut, and the potential for off-target effects on beneficial bacteria.

Combination therapies with biologics may yield synergistic benefits. Patients with severe, steroid-resistant asthma often receive anti-IL-5 or anti-IL-4Rα biologics. If gut dysbiosis contributes to persistent type 2 inflammation, combining a biologic with a microbiome-targeted intervention could improve response rates or allow dose reduction. This hypothesis has not yet been tested (168). However, the biological rationale is strong: biologics address downstream inflammation, while microbiome-targeted interventions address upstream drivers. Combining the two approaches could provide more comprehensive disease modification than either alone (165).

### 5.3 Practicalities of delivery, manufacturing, and quality control

Translating live microbial therapeutics from bench to bedside requires careful attention to practical considerations often overlooked in academic research. Three interconnected challenges—manufacturing, delivery, and quality control—demand particular focus. Manufacturing of live biotherapeutics presents substantial technical and financial hurdles: oxygen-free cultivation, formulation science for stabilization (lyophilization, enteric coatings), and compliance with Good Manufacturing Practice (GMP) are all essential. The cost of GMP-grade production is substantial and poses a particular barrier to academic-led trials, while rigorous analytical characterization—whole-genome sequencing, resistome assessment, and potency assays—is equally critical for product consistency and regulatory approval.

Delivery poses distinct challenges, particularly for postbiotics and metabolites. Oral butyrate is rapidly absorbed in the upper gastrointestinal tract and may not reach the colon in sufficient concentrations. Tributyrin, a prodrug, partially overcomes this limitation but still requires enteric coating to prevent premature release. Nanotechnology-enabled delivery strategies are being explored to target metabolites to the lower gastrointestinal tract or even directly to the lung (169, 170); these approaches are promising yet remain at an early stage. Quality control adds another layer of complexity beyond that of conventional drugs. Live products must maintain viability throughout the supply chain, while metabolite products must demonstrate stability and consistent composition across batches. Regulatory standards are still evolving, and manufacturers must stay abreast of changing requirements (169).

### 5.4 Ethical, legal, and social implications

Microbiome-targeted therapies raise ethical, legal, and social considerations that demand proactive attention. Early-life interventions require long-term safety monitoring; the microbiome’s lifelong role in immune education means developmental windows carry risks of delayed consequences (171). Registries and extended follow-up are essential. Data governance is equally critical: microbiome profiles are personally identifiable and dynamically reveal diet, medication, and disease status, unlike stable genetic data (172). Clear guidelines for sharing, consent, and result return are urgently needed. Health equity cannot be overlooked; high costs may limit access to affluent populations unless cost-reduction strategies and equitable deployment are prioritized (173). Finally, commercialization raises ownership and benefit-sharing questions around donor microbiomes as shared resources—issues that must be addressed early, not belatedly.

### 5.5 Challenges and future directions

Several scientific and clinical challenges must be overcome before microbiome-targeted therapies can realize their potential (**Figure 2**). Inter-individual variability poses a fundamental hurdle: baseline microbiome composition, genetics, diet, and environmental exposures all shape treatment response, making biomarker-based enrichment essential for identifying patients most likely to benefit. Ecological resilience of microbial communities presents another obstacle—after therapy withdrawal, the gut ecosystem often reverts to its original dysbiotic state, suggesting that chronic or repeated dosing may be necessary, with attendant cost and safety implications. Combining microbial modulation with dietary changes that support the desired community state may offer an alternative path to durability.

**Figure 2:**
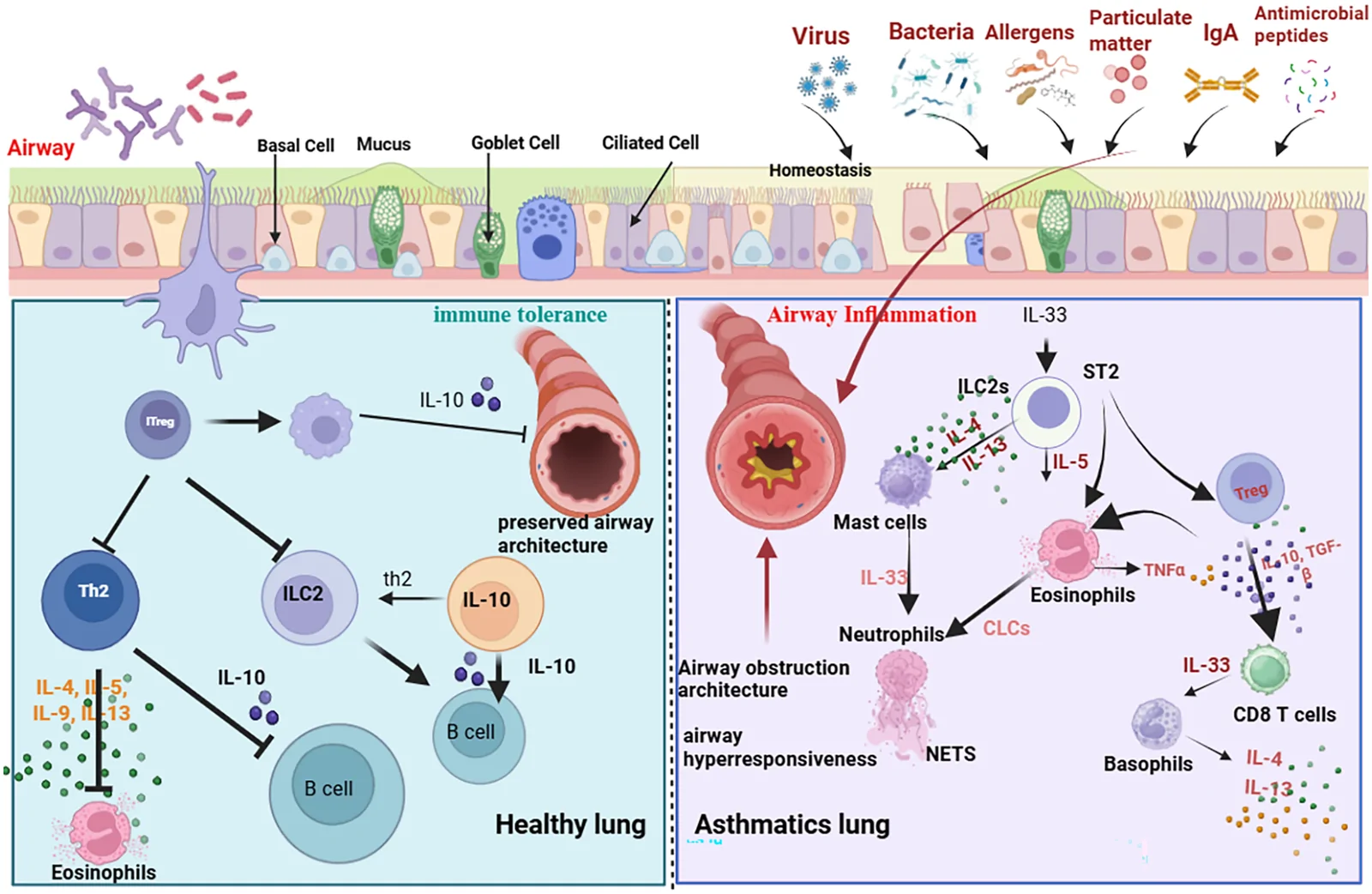
Immune regulation in the healthy versus asthmatic lung. The diagram illustrates the critical differences in immune homeostasis and inflammatory dysregulation between the healthy airway (left) and the asthmatic airway (right). In the healthy lung (homeostasis), the airway epithelium (composed of basal, goblet, and ciliated cells) provides a physical and immunological barrier against inhaled pathogens, allergens, and particulates, supported by mucus, IgA, and antimicrobial peptides. Induced regulatory T cells (iTregs) maintain immune tolerance by secreting IL-10, which suppresses airway inflammation, while directly inhibiting type 2 immune responses: they block Th2 cell activation (preventing IL-4, IL-5, IL-9, and IL-13 production), restrain ILC2 activity, and limit eosinophil and B cell effector functions, preserving normal airway architecture and function. In the asthmatic lung, barrier disruption and chronic inflammation drive pathological type 2 responses. Epithelial cells release the alarmin IL-33, which activates ILC2s via the ST2 receptor. Activated ILC2s produce IL-5, IL-4, and IL-13, triggering eosinophil expansion, mast cell and basophil activation. Eosinophils release pro-inflammatory cytokines (TNF-α) and form cytolytic leukocyte complexes (CLCs), while neutrophils release extracellular traps (NETs), amplifying tissue damage and airway remodeling. Th2 cells and basophils further sustain inflammation via IL-4 and IL-13, leading to airway hyperresponsiveness and asthma pathology.

Preclinical models remain a significant limitation. Most studies rely on defined-diet, specific-pathogen-free mice that poorly recapitulate human microbiome complexity and dietary variation (174, 175). Humanized gnotobiotic mice and organoid systems offer incremental improvements yet still fall short, leaving the translational gap between mouse models and human disease as a major barrier. Clinical trial design requires equally careful consideration. Adaptive designs with biomarker-based enrichment are preferable to traditional fixed designs, and mechanistic endpoints—changes in SCFA levels or Treg frequency, for example—can provide early evidence of target engagement while guiding dose selection. Patient-reported outcomes, including asthma control and quality of life, remain essential for demonstrating meaningful clinical benefit. Finally, regulatory pathways for novel modalities are still evolving; live biotherapeutics, eLBPs, and phage therapies fall outside traditional drug categories and may require new frameworks, making early engagement with regulators critical for successful development.

### 5.6 A proposed clinical algorithm

Integrating the above insights, we propose a staged algorithm for microbiome-informed asthma management. Although hypothetical and requiring prospective validation in endotype-stratified trials, this framework offers a structured approach for integrating microbiome diagnostics and therapeutics into clinical practice.

Patient selection focuses on those with severe or treatment-refractory asthma—individuals with the greatest unmet need and highest likelihood of benefiting from novel approaches. Selected patients then undergo diagnostic profiling via stool or serum metabolomics (SCFAs, tryptophan metabolites, bile acids) and/or microbial signature analysis (e.g., Faecalibacterium abundance), with the choice of method guided by cost, availability, and therapeutic options. Profiling results enable endotype stratification—for instance, “SCFA-deficient eosinophilic asthma” (low butyrate, low Faecalibacterium, elevated eosinophils) or “fungal-associated severe asthma” (elevated β-glucan, Candida abundance). Targeted interventions follow accordingly: high-fiber diet, tributyrin, or SCFA-producing consortia for the former; antifungal or barrier-restorative therapies for the latter. Response is monitored through repeat metabolite measurement and clinical outcomes (Asthma Control Questionnaire, FeNO, exacerbation rate), with non-responders considered for alternative or combination approaches (**Figure 3**).

**Figure 3:**
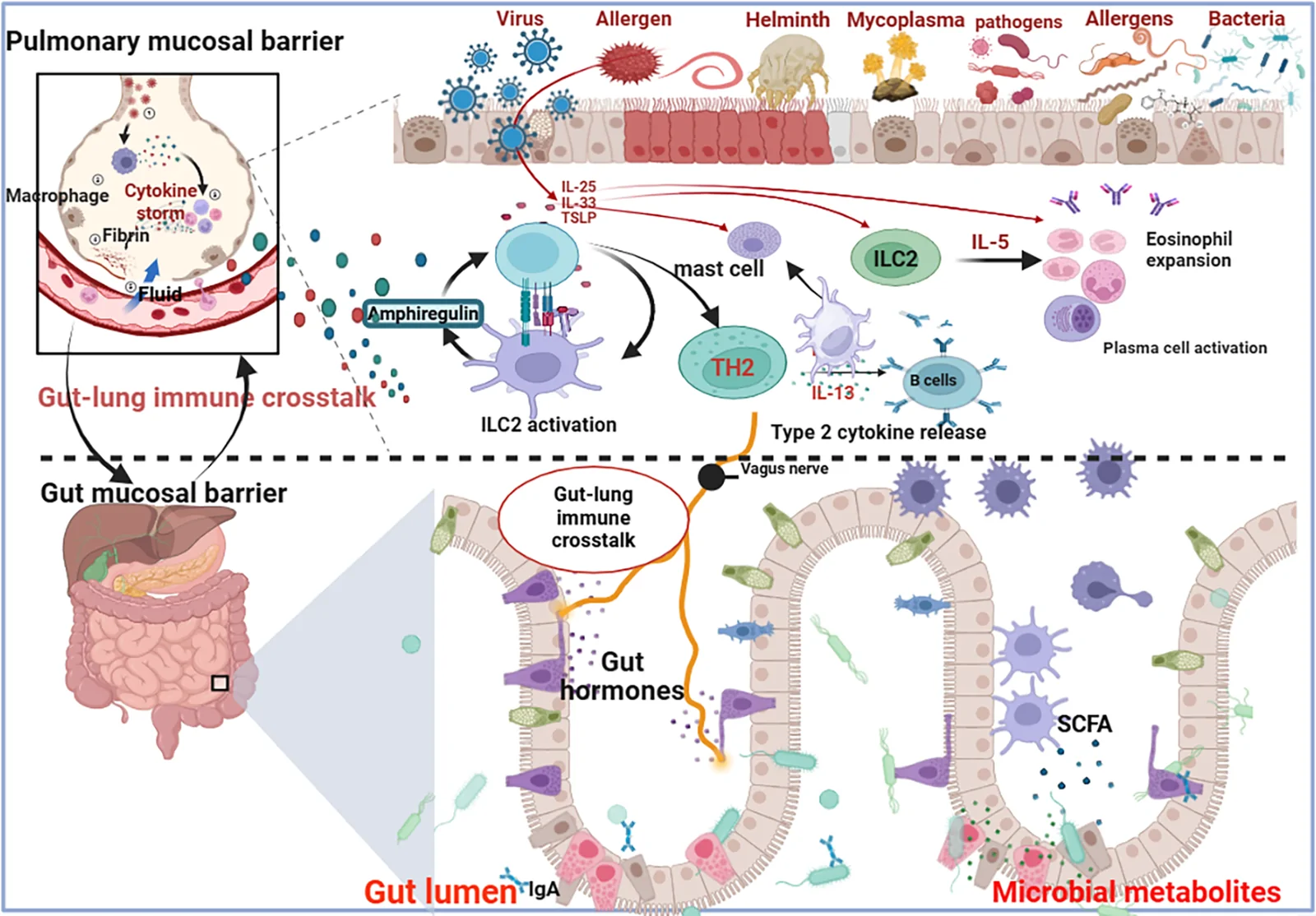
Mechanistic pathways of gut-lung immune communication. At mucosal barriers, allergens, helminths, or viruses trigger the release of epithelial-derived cytokines (IL-25, IL-33, TSLP), activating group 2 innate lymphoid cells (ILC2s) and type 2 helper T (TH2) cells. These cells secrete type 2 cytokines (IL-4, IL-5, IL-13) that drive eosinophil expansion, plasma cell activation, and mast cell responses, amplifying type 2 inflammation. Amphiregulin release further supports epithelial repair and tissue homeostasis. Gut-derived signals shape both local and systemic immunity. The vagus nerve and gut hormones provide neuro-immune communication, while microbial metabolites (including short-chain fatty acids, SCFAs) and commensal bacteria modulate epithelial barrier function, IgA production, and immune cell activity in the gut lumen and lamina propria. Together, these gut-derived signals traffic via systemic circulation to influence pulmonary immunity, forming the basis of gut-lung immune crosstalk.

Cost remains a major implementation barrier, with comprehensive multi-omics profiling currently priced at several hundred to over a thousand US dollars per patient. Until automation, multiplexing, and economies of scale drive prices down, adoption will likely be confined to specialized centers and trials. Technological democratization—through targeted, low-cost assays such as fecal SCFA quantification or point-of-care enzymatic tests—will be essential for widespread clinical adoption.

## 6 Conclusion and future perspectives

The gut–lung axis reframes asthma as a systemic, ecology-informed disorder. Remote microbial communities shape immune set-points, determine the balance between tolerance and hypersensitivity, and influence susceptibility to inflammatory exacerbations and structural remodeling. This review has synthesized evidence across microbial ecology, metabolite-specific immune reprogramming, causal inference frameworks, and precision therapeutic pipelines.

Several major barriers impede clinical translation. Establishing causality in humans remains the most formidable obstacle. Most human studies are observational. Randomised controlled trials can establish intervention–outcome links, yet definitive molecular causality demands triangulation across genetic, mechanistic, and interventional evidence. Methodological heterogeneity also poses a significant challenge. Lack of standardization in sample collection, sequencing, and bioinformatic pipelines limits cross-study comparability. Antibiotic and corticosteroid use profoundly disrupts gut microbial diversity and confounds association studies. High inter-individual variability—driven by diet, geography, age, genetics, and concomitant medications—ensures that no single microbial signature or therapeutic strategy will apply to all patients.

To bridge the bench-to-bedside gap, future research must pursue several priority directions in parallel. Establishing causality will require gnotobiotic models, multi-organ chips, and humanized mice—platforms that enable controlled testing of specific microbial functions and their pulmonary effects. These tools, combined with genetic approaches such as Mendelian randomization, can distinguish causal drivers from epiphenomena. Transitioning from descriptive to predictive science demands integration of metagenomics, metabolomics, and proteomics with longitudinal sampling; function-centric modeling that emphasizes metabolic pathways over taxonomic composition will enhance reproducibility and mechanistic insight. Expanding beyond bacteria is equally urgent—fungi, viruses, and protozoa have been largely overlooked yet likely contribute meaningfully to asthma pathogenesis, and cross-domain interactomics represents a frontier ripe for therapeutic discovery. Rigorous clinical trials with deep multi-omics profiling and biomarker-based enrichment are essential for validating causal hypotheses, with mechanistic endpoints built in to confirm target engagement and identify responders. Finally, standardized protocols for sample collection, sequencing, and bioinformatics must be developed to enable cross-study comparability and meta-analyses—a goal that demands coordinated community action.

The translational promise is profound. Reliably identifying patients whose disease is driven by specific microbial functional deficits would be a major advance. So would restoring missing or dysfunctional community capabilities through targeted nutritional or microbial interventions. Modulating immune trafficking and metabolite signaling at the right developmental window could move us from symptom control to durable disease modification—and even primary prevention. Realizing this promise requires precision across multiple dimensions: diagnostics, nutrition, therapeutics, timing, and evaluation.

The technology to implement these elements is now mature. High-throughput sequencing, metabolomics, gnotobiotic models, and synthetic biology have advanced sufficiently for deployment in clinical research. The remaining obstacles are foundational. Establishing causality in humans with the same rigor achieved in animal models remains a core challenge. So does building standardized, generalizable biomarkers that perform across diverse populations and geographies. Ensuring long-term safety and ecological stewardship of live microbial therapeutics is equally critical. Finally, regulatory and health system pathways must evolve to accommodate these novel modalities.

Shifting the therapeutic focus from managing inflammation in the lung to restoring microbial ecology in the gut places us on the brink of a transformative era in asthma management. The path ahead is neither simple nor risk-free, yet it is increasingly clear. Coupling rigorous mechanistic science with careful clinical translation—and a commitment to ethical and equitable deployment—will allow the microbiome to move from compelling biological insight to a platform for precision immunomodulation in asthma.

## Glossary

AhR: Aryl hydrocarbon receptor
cAMP: Cyclic adenosine monophosphate
CCR9: C-C chemokine receptor type 9
DC: Dendritic cell
DCA: Deoxycholic acid
eLBP: Engineered live biotherapeutic product
ERK: Extracellular signal-regulated kinase
EV: Extracellular vesicle
FeNO: Fractional exhaled nitric oxide
FEV1: Forced expiratory volume in one second
FMT: Fecal microbiota transplantation
Foxp3: Forkhead box protein P3
FXR: Farnesoid X receptor
GALT: Gut-associated lymphoid tissue
GMP: Good Manufacturing Practice
GPCR: G-protein coupled receptor
GPR: G-protein coupled receptor
HDAC: Histone deacetylase
I3A: Indole-3-aldehyde
IDO: Indoleamine-2,3-dioxygenase
IgE: Immunoglobulin E
IL: Interleukin
ILC: Innate lymphoid cell
IPA: Indole-3-propionic acid
LCA: Lithocholic acid
mbQTL: Microbial quantitative trait locus
MEK: Mitogen-activated protein kinase kinase
MHC: Major histocompatibility complex
MOFA: Multi-Omics Factor Analysis
MR: Mendelian randomization
MRS: Microbiome risk score
NF-κB: Nuclear factor kappa-light-chain-enhancer of activated B cells
NOD2: Nucleotide-binding oligomerization domain-containing protein 2
PKA: Protein kinase A
PSA: Polysaccharide A
PXR: Pregnane X receptor
RCT: Randomized controlled trial
S1PR1: Sphingosine-1-phosphate receptor 1
SCFA: Short-chain fatty acid
SOCS3: Suppressor of cytokine signaling 3
TCA: Tricarboxylic acid
TGR5: Takeda G-protein-coupled receptor 5
Th: T helper cell (subtypes, Th2, Th17)
TLR: Toll-like receptor (e.g., TLR2)
TMLE: Targeted maximum likelihood estimation
Treg: Regulatory T cell
TSLP: Thymic stromal lymphopoietin
